# Assessment of human adipose‐derived stem cell on surface‐modified silicone implant to reduce capsular contracture formation

**DOI:** 10.1002/btm2.10260

**Published:** 2021-10-08

**Authors:** Chanutchamon Sutthiwanjampa, Byung Ho Shin, Na Eun Ryu, Shin Hyuk Kang, Chan Yeong Heo, Hansoo Park

**Affiliations:** ^1^ School of Integrative Engineering, Chung‐Ang University Seoul Republic of Korea; ^2^ Department of Biomedical Engineering College of Medicine, Seoul National University Seoul Republic of Korea; ^3^ Department of Plastic and Reconstructive Surgery Chung‐Ang University Hospital Seoul Republic of Korea; ^4^ Department of Plastic and Reconstructive Surgery Seoul National University Bundang Hospital Seongnam Republic of Korea; ^5^ Interdisciplinary Program for Bioengineering College of Engineering, Seoul National University Seoul Republic of Korea; ^6^ Department of Plastic and Reconstructive Surgery College of Medicine, Seoul National University Seoul Republic of Korea

**Keywords:** antifibrotic property, capsular contracture, foreign body response, human adipose‐derived stem cells, itaconic acid, poly(dimethylsiloxane), surface conjugation

## Abstract

Medical devices made from poly(dimethylsiloxane) (PDMS)‐based silicone implants have been broadly used owing to their inert properties, biocompatibility, and low toxicity. However, long‐term implantation is usually associated with complications, such as capsular contracture due to excessive local inflammatory response, subsequently requiring implant removal. Therefore, modification of the silicone surface to reduce a risk of capsular contracture has attracted increasing attention. Human adipose‐derived stem cells (hASCs) are known to provide potentially therapeutic applications for tissue engineering, regenerative medicine, and reconstructive surgery. Herein, hASCs coating on a PDMS (hASC‐PDMS) or itaconic acid (IA)‐conjugated PDMS (hASC‐IA‐PDMS) surface is examined to determine its biocompatibility for reducing capsular contracture on the PDMS surface. In vitro cell cytotoxicity evaluation showed that hASCs on IA‐PDMS exhibit higher cell viability than hASCs on PDMS. A lower release of proinflammatory cytokines is observed in hASC‐PDMS and hASC‐IA‐PDMS compared to the cells on plate. Multiple factors, including in vivo mRNA expression levels of cytokines related to fibrosis; number of inflammatory cells; number of macrophages and myofibroblasts; capsule thickness; and collagen density following implantation in rats for 60 days, indicate that incorporated coating hASCs on PDMSs most effectively reduces capsular contracture. This study demonstrates the potential of hASCs coating for the modification of PDMS surfaces in enhancing surface biocompatibility for reducing capsular contracture of PDMS‐based medical devices.

## 1 INTRODUCTION

Poly(dimethylsiloxane) (PDMS)‐based silicone implants have been used in breast augmentation, rhinoplasty, and postmastectomy reconstruction in the plastic and reconstructive surgery fields for several decades. Recipients are usually satisfied with the tissue‐like mechanical properties of silicone‐based implants; however, their limited biocompatibility poses a challenge. For example, the surfaces of silicone breast implants have substantial limitations owing to the formation of a constrictive fibrotic capsule (known as capsular contracture) following implantation, which in addition to esthetic malfunction, has been shown to cause illness and deformation.^1^ It has been reported that capsular contracture occurs over time ranging from months to years postimplantation.^2^, ^3^, ^4^, ^5^ Capsular contracture has been hypothesized to be caused by excessive foreign body reactions on silicone surfaces. Notably, capsule formation is a normal response to foreign bodies, whereas contracture is not. The formation of capsular contracture seems to be a multifactorial process. Immunological reactions of the patient to foreign bodies due to silicone gel leakage, dust, or powdered gloves, exaggerated inflammatory responses to foreign prosthetic materials, and bacterial inoculation and biofilm formation within the implant have been proposed as pathomechanisms underlying capsular contracture.^6^, ^7^, ^8^, ^9^ To overcome these challenges, current research has focused on the surface modification of prosthetic materials. A recent study reported the potential use of surface‐modified implants to reduce capsular fibrosis via a local antifibrotic effect based on modifying the surface of silicone implants with halofuginone, an antifibrotic drug; this implant was found to decrease host responses to foreign bodies.^10^ The areas of focus in research on the modification of silicone implant surfaces include increasing the hydrophilicity and biocompatibility of the surface and reducing excessive host reactions to foreign bodies. The surface wettability of any polymer can be improved by increasing its hydrophilicity, thereby enhancing its biocompatibility.^11^

Among the methods used to prepare biocompatible surfaces, surface coating with biomembrane‐mimicking materials is considered the most desirable. Previously, successfully synthesized biomembrane‐mimicking polymers with various phospholipid head groups have been reported.^12^ These new biomembrane‐mimicking polymers could be used in various application platforms in biomedical fields, such as tissue engineering or bioimplantation, in the near future. Itaconic acid (IA) is an organic compound known to be microbe‐resistant, chemically reactive, biodegradable, biocompatible, and nontoxic.^13^ It has been suggested to have excellent potential in a wide range of scientific fields, such as agricultural, food, pharmaceutical, biomedical, and other industries.^14^ The two carboxyl groups of IA contribute to its hydrophilic property. IA has applications in wound healing, coat formation, water uptake, drug transport, and hydrogel forming^15^, ^16^, ^17^, ^18^, ^19^; in addition, it blocks isocitrate lyase, the primary enzyme of the glyoxylate shunt, a key pathway for bacterial growth under specific conditions.^20^ In our recent studies, PDMS conjugated with 150 mM IA and 0.50 wt% IA‐gelatin polymer demonstrated excellent and effective anti‐protein adhesion, antibacterial adhesion, and in vivo anti‐fibrotic functions.^21^, ^22^

In recent years, various stem cells, such as T cells, hematopoietic stem cells, human adipose‐derived stem cells (hASCs), and induced pluripotent stem cells, have attracted considerable attention regarding their applications in biomedical fields and have been favorable candidates for regenerative medicine, cell therapy, and cell engineering.^23^ Among the stem cells using in biomedical therapy, hASCs have gained great interest in cell‐based therapeutic applications in regenerative medicine and tissue engineering.^24^ These stem cells can differentiate along multiple mesodermal, myogenic, and nonmesodermal lineages, such as adipogenic, osteogenic, muscle, and epithelial cells.^25^ The isolation of hASCs from a stromal vascular fraction of adipose tissue is relatively easier than the isolation of other stem cells, and hence, small amounts of adipose tissue can yield a large number of stem cells compared with that from other sources.^25^ In particular, hASCs are a miscellaneous population of cells and owing to their inherent multipotency and ability to enhance vascularization and adipogenesis, which makes them superior to other materials, they are known to have a broad range of potential therapeutic applications and thus represent a favorable cell‐based therapeutic tool for tissue engineering, regenerative medicine, and reconstructive surgery.^26^, ^27^ Based on these findings, autologous adipose tissue or adipose tissue with hASCs has been used for breast reconstruction in patients with breast cancer who have undergone mastectomy.^28^, ^29^ Moioli et al. reported that hASCs regenerated functional, highly vascularized adipose tissue following transplantation in a murine xenograft model.^30^ Additionally, hASCs have been reported to possess important immunoregulatory effects via paracrine signaling,^31^ including immunosuppressive effects on several immune cells under varying conditions,^32^, ^33^ and protect tissue against ischemia–reperfusion injury, thereby increasing tissue survival.^25^ Moreover, hASCs have been applied to various medical devices to enhance biocompatibility for application in many medical fields, such as dental implants^23^ and osteogenesis.^34^, ^35^ Many alternative approaches have focused on using hASCs to reduce fibrosis and capsular contracture over the last decades.^36^, ^37^, ^38^, ^39^ Recently, Thomé et al. found that hASC‐enriched fat grafting could successfully reduce capsular contracture formation in rats.^40^ Further, various silicone surface modification techniques have been used to reduce the complications from silicone‐based medical devices.^23^, ^34^, ^41^, ^42^, ^43^, ^44^, ^45^ Nevertheless, few studies have researched silicone implant surface modification using hASCs to reduce breast capsular contraction. Barr et al. reported that a biomimetic breast adipose tissue‐derived breast implant surface could effectively decrease the inflammatory phase of the implant‐driven foreign body reaction related to capsular contracture in vitro.^36^ However, to the best of our knowledge, no previous research has investigated the surface modification of silicone implants using hASCs to reduce capsular contracture in vivo. We therefore used hASCs, according to their multipotency, to modify a PDMS surface by coating them on the surface of bare PDMS or IA‐conjugated PDMS (IA‐PDMS), a hydrophilic modified‐surface developed in our previous studies.^21^, ^22^ We aimed to evaluate and compare the ability of IA‐PDMS, hASC‐coated PDMS (hASC‐PDMS), and hASC‐coated IA‐PDMS (hASC‐IA‐PDMS) to reduce the formation of capsular contracture. We hypothesized that hASC‐IA‐PDMS could most efficiently reduce the formation of capsular contracture following silicone implantation.

## 2 RESULTS AND DISCUSSION

### 2.1 Preparation of IA‐PDMS

**FIGURE 1:**
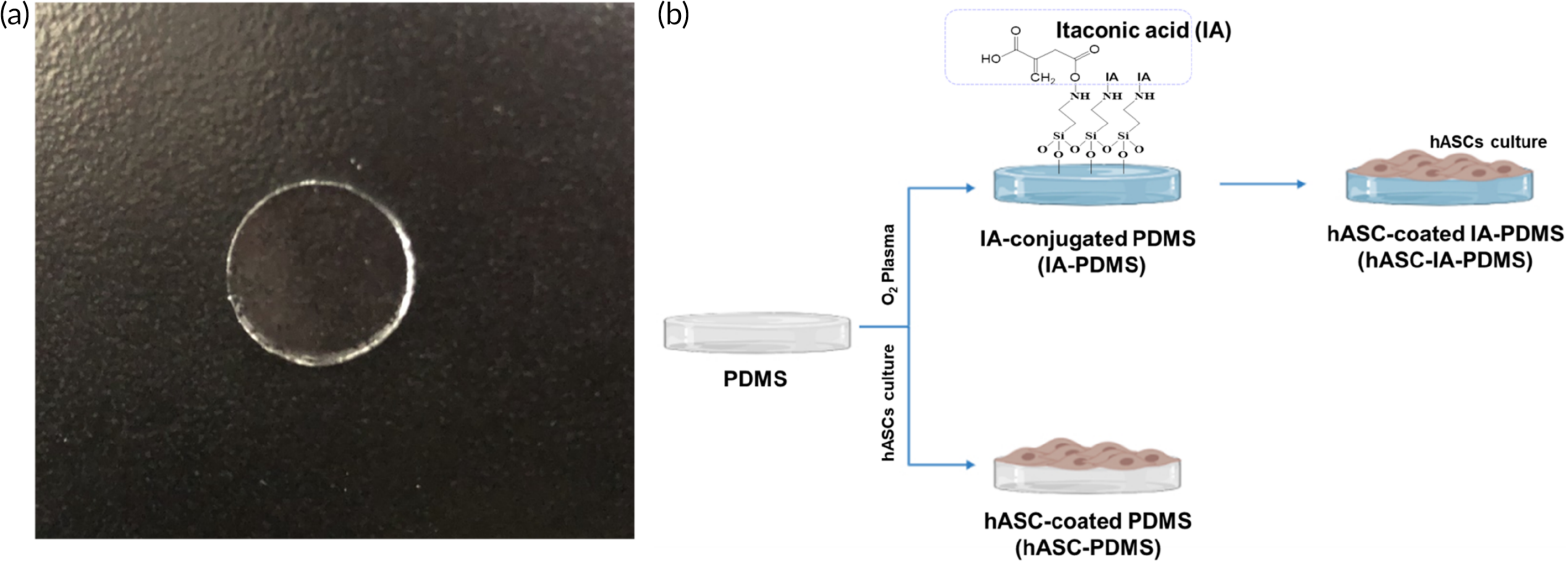
(a) Poly(dimethylsiloxane) (PDMS) membrane samples. (b) Schematic representation of the coating of a PDMS surface with human adipose‐derived stem cells (hASCs), as well as PDMS‐conjugated itaconic acid (IA) (150 mM) coating with hASCs

PDMS was prepared by mixing the base and curing agent at 10:1 (w/w), which was reported as the best ratio for biological application^46^, ^47^ and has been used as a model of silicone implants in several studies.^48^, ^49^, ^50^, ^51^, ^52^ We chemically modified the surface of PDMS with 150 mM IA (Figure 1). This concentration was selected because previously, it was reported to result in the excellent enhancement of biocompatibility and reduction in capsular contracture formation of the PDMS surface, similar to results with the 0.50 wt% IA‐GT polymer but with an easier preparation method than the latter.^21^, ^22^ Measurement of the water contact angle and the attenuated total reflectance/Fourier transform infrared (ATR/FTIR) spectra was used to confirm the formation of IA on the PDMS surface. As shown in Figure S1a, the contact angle was significantly decreased from 96.74 ± 5.998° to 35.66 ± 3.552° following IA conjugation (*p* < 0.0001), indicating an improvement in the wettability of the PDMS surface, resulting in hydrophilicity owing to the presence of IA. Additionally, this was confirmed in Figure S2a, which illustrates the formation of IA based on the appearance of the peak at 1650 cm^−1^ and 1548 cm^−1^, indicating C═O and C—N groups, respectively. More specifically, C═O stretching was introduced by IA, whereas the C—N bonding indicates the formation of an amide.^21^

### 2.2 Characterization of human adipose‐derived stem cells and investigation of cell viability, morphology, and adhesion patterns

**FIGURE 2:**
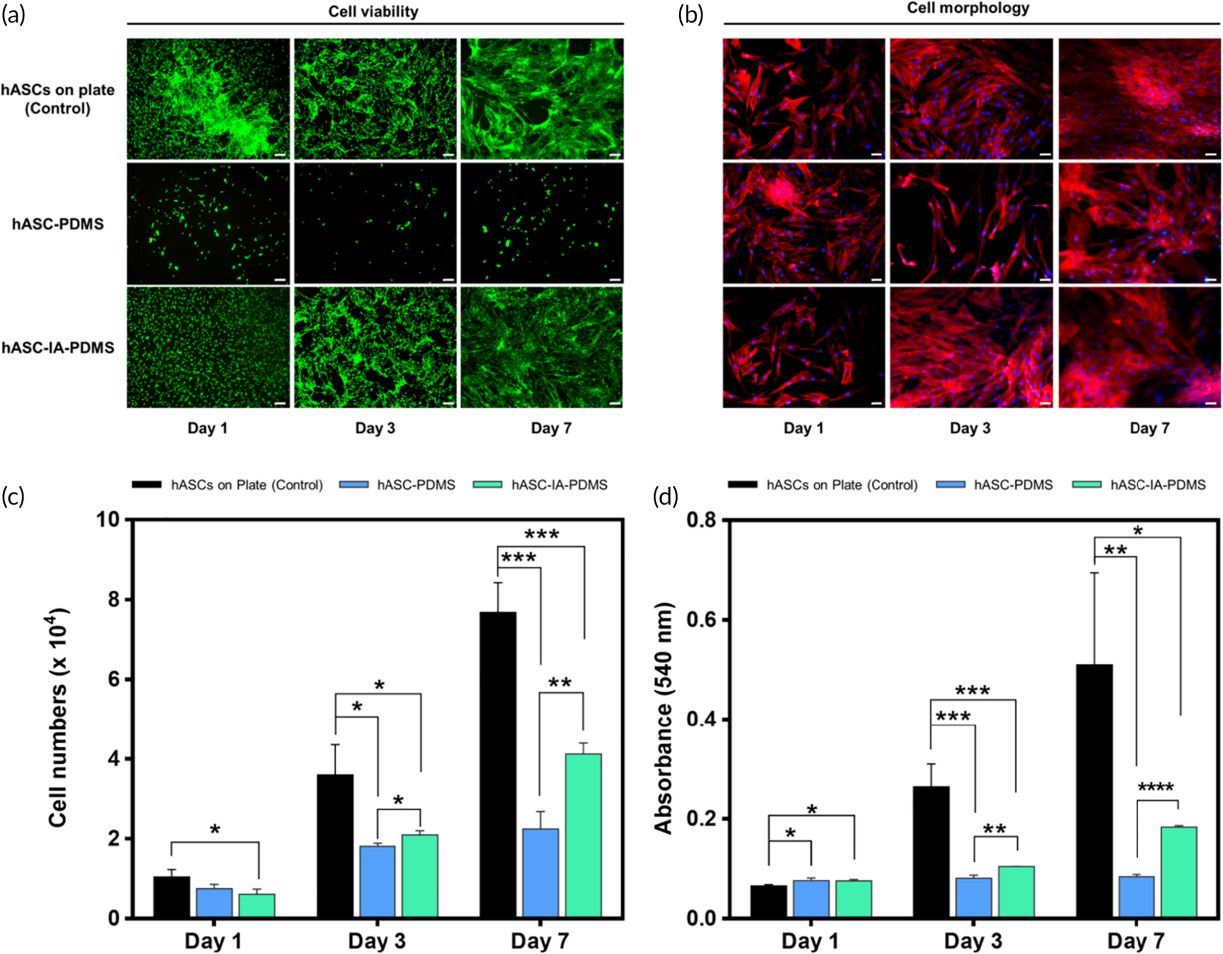
(a) Fluorescence micrograph of in vitro cell viability and (b) cell morphology of human adipose‐derived stem cells (hASCs) on a plate, hASC‐poly(dimethylsiloxane) (PDMS), and hASC‐itaconic acid (IA)‐PDMS at 1, 3, and 7 days at a magnification of 4× (for cell viability) or 10× (for cell morphology). Results of (c) cell proliferation and (d) cell cytotoxicity from the CCK‐8 and MTT assay, respectively, for hASCs cultured on a culture plate, PDMS, and IA‐PDMS for 1, 3, and 7 days. (e) Scanning electron microscopy (SEM) micrographs of hASCs on plates, PDMS, IA‐PDMS, hASC‐PDMS, and hASC‐IA‐PDMS at a magnification of 100×. hASCs at Day 3 were used in this evaluation. Data are shown as the mean ± SD (*n* = 3). *p < 0.05, **p < 0.01, ***p < 0.001, and ****p < 0.0001 (one‐way ANOVA, Bonferroni)

We then characterized the phenotype of hASCs via fluorescence‐activated cell sorting analysis in accordance with the low expression of surface makers including CD14, CD34, and CD45 and high expression of major surface marker including CD73 and CD105.^53^, ^54^, ^55^, ^56^ As predicted, high expression of CD73 and CD105 and low expression of CD14, CD34, and CD45 were observed (Figure S3), consistent with that in prior studies.^53^, ^57^, ^58^ To observe cell viability and the adhesion pattern of hASCs grown on a culture plate, PDMS, and IA‐PDMS, the Live/Dead assay was used. This assay showed that the viability of cells on IA‐PDMS surfaces was better than that on a PDMS surface and comparable to that on a culture plate (control) at 1, 3, and 7 days (Figure 2a). We also observed the morphology of cells grown on a culture plate, PDMS, and IA‐PDMS using rhodamine/DAPI (4',6‐diamidino‐2‐phenylindole) staining. Accordingly, the morphology of cells cultured on an IA‐PDMS surface was similar to that on a culture plate at 1, 3, and 7 days (Figure 2b). However, the morphology of cells cultured on PDMS surfaces appeared more round‐shaped than those grown on IA‐PDMS and a culture plate (Figure 2b).

The results of a Cell Counting Kit‐8 (CCK‐8) assay demonstrated the proliferation of hASCs on the culture plate, PDMS, and IA‐PDMS at 1, 3, and 7 days (Figure 2c). Cell numbers on IA‐PDMS were significantly lower than those on culture plates (control) at 1, 3, and 7 days (*p* < 0.05), whereas those on PDMS were significantly lower than those on the culture plate at 3 and 7 days (*p* < 0.05). However, although cell proliferation on IA‐PDMS was significantly decreased relative to that on the culture plate, it remained significantly increased compared to that on PDMS on Days 3 and 7 (*p* < 0.05). The increased cell proliferation on IA‐PDMS might be attributed to the hydrophilicity of the IA‐PDMS surface, which might have inhibited protein adsorption and subsequent cell adhesion. These results were consistent with those of previous studies, which showed that hydrophilic surfaces were better at preventing protein adsorption than hydrophobic surfaces.^21^, ^50^, ^59^, ^60^ Figure 2d shows cell cytotoxicity results using the 3‐[4,5‐dimethylthiazol‐2‐yl]‐2,5‐diphenyltetrazolium bromide (MTT) assay. The PDMS and IA‐PDMS samples showed significantly lower absorbance than cells on a plate at Days 1, 3, and 7 (*p* < 0.05). However, we observed increases in the absorbance of PDMS and IA‐PDMS from Days 1 to 7, with that of IA‐PDMS being significantly higher than that of PDMS (*p* < 0.05). This finding confirmed that cells were viable and capable of proliferation.

### 2.3 Stability of IA‐PDMS surface

Stability of the IA‐PDMS surface was examined for up to 60 days using the water contact angle and FTIR as parameters of surface hydrophilicity stability and in vitro anti‐protein adsorption, antibacterial adhesion, and cell studies as parameters of surface biocompatibility. IA‐PDMS could maintain surface hydrophilicity (Figures S1b,c and S2b), as well as surface biocompatibility (Figures S4–S6), even after exposure to the air for up to 60 days. After storage in de‐ionized water (DI) and Dulbecco's phosphate‐buffered saline (DPBS), IA‐PDMS showed better hydrophilic stability and biocompatibility than when exposed to the air. Since the in vivo environment is hydrophilic, for this modified surface, it was considered that biocompatibility in vivo was improved and could be maintained for up to 60 days. Similar results were obtained in our previous study, which showed that the hydrophilicity and biocompatibility of an oxygen (O_2_) plasma‐treated silicone implant surface could be maintained for up to 60 days in DI storage.^61^ However, preserving a silicone implant in DI before implantation might not represent proper, realistic use, and O_2_ plasma‐treated implants showed unstable properties upon exposure to the air. Therefore, IA‐PDMS surface modification would be a better choice for improving silicone implant surfaces, because hydrophilicity and biocompatibility could be maintained even after exposure to the air.

### 2.4 Surface morphologies

Figure 2e shows the surface morphologies of PDMS and IA‐PDMS, as well as those of hASCs on a cultured plate, hASC‐PDMS, and hASC‐IA‐PDMS using scanning electron microscopy (SEM). The surfaces of PDMS and IA‐PDMS were smooth, even, and clear, consistent with the results of a previous study.^21^ Cells on IA‐PDMS were shown to be long and widely spread on the sample surface with a copious amount of cells, exhibiting the same pattern as those on a culture plate. In contrast, cells on the PDMS surface were spherical, and their number was far lower than that on the culture plate and IA‐PDMS. The difference observed in cell morphology between the PDMS and IA‐PDMS surfaces was attributed to the hydrophilicity of the material surfaces. A hydrophilic surface is assumed to allow cells to better attach to the surface, resulting in a long and flattened cell morphology. In contrast, a hydrophobic surface would provide smaller attachment areas on its surface, resulting in a more rounded cell morphology.^62^, ^63^

### 2.5 In vitro cytokine release

**FIGURE 3:**
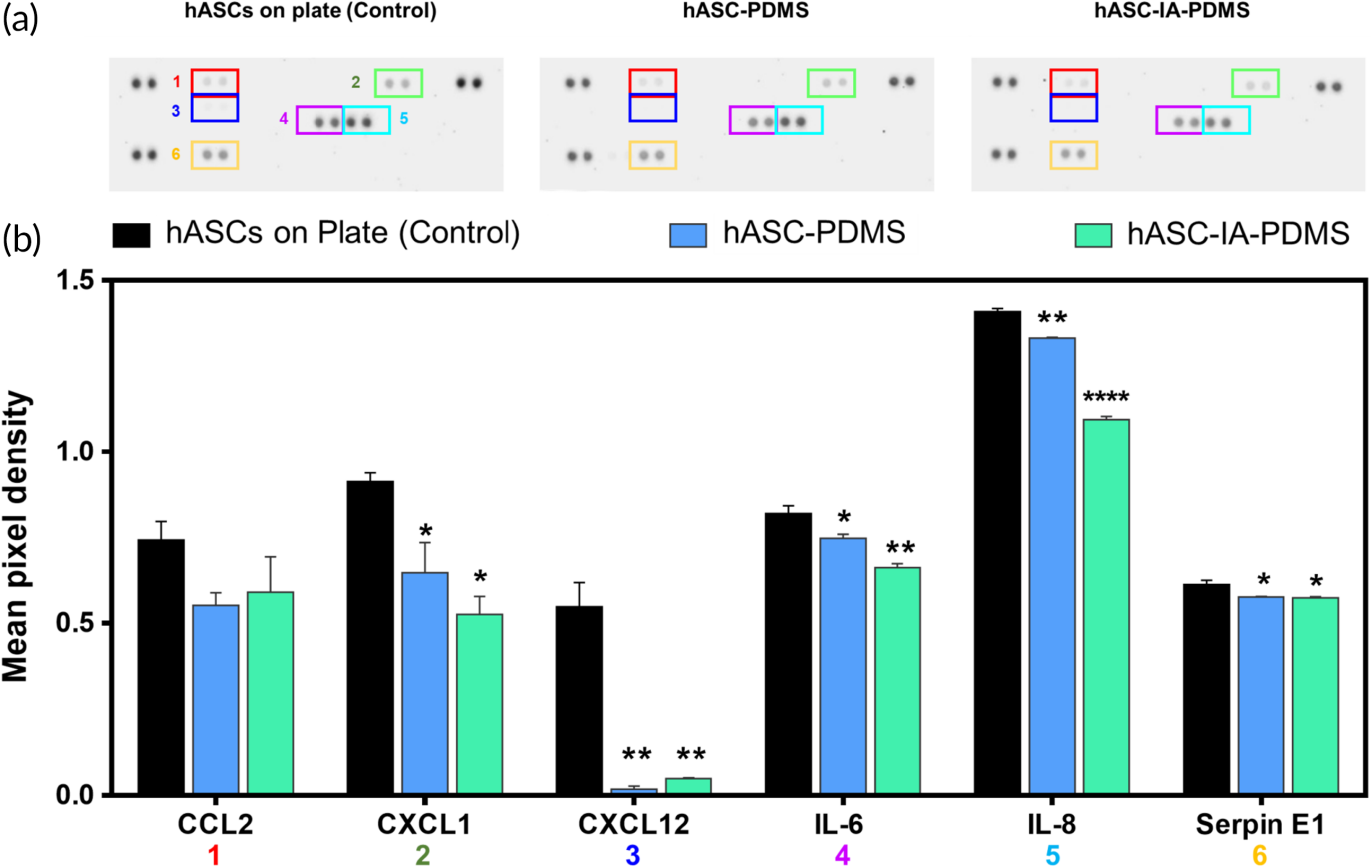
(a) Cytokine array analysis and (b) quantitative cytokine array analysis of cytokine release from human adipose‐derived stem cells (hASCs) cultured on cell culture plates, poly(dimethylsiloxane) (PDMS), and IA‐PDMS. hASCs at Day 3 (without changing media) were used for this evaluation. Data are shown as the mean ± SD (*n* = 2). *p < 0.05, **p < 0.01, and ***p < 0.0001, ****p < 0.0001 (one‐way ANOVA, Bonferroni)

We screened for in vitro cytokine release using a proteome array and showed that the identified factors included the chemokine ligand (CCL)2, chemokine (C‐X‐C motif) ligand CXCL12, CXCL1, interleukin (IL)‐6, IL‐8, and endothelial plasminogen activator inhibitor (SERPINE1) (Figure 3). We hence noted that the cytokines released from hASCs were similar to those reported in a previous study,^64^ but we observed no significant differences between the PDMS (control) and hASC‐coated samples (hASC‐PDMS and hASC‐IA‐PDMS; *p* < 0.05); however, the hASC‐coated groups showed a lower pixel density for CCL2 than the control. Similarly, the pixel densities of CXCL1 and CXCL12 from hASC‐PDMS and hASC‐IA‐PDMS were shown to be significantly lower than those of the control (*p* < 0.01). The pixel density values of IL‐6 from the hASC‐coated samples were also significantly lower than those of the control (*p* < 0.05), and the same was true for IL‐8 (*p* < 0.01) and SERPIN E1 (*p* < 0.05).

The CCL2 chemokine, associated with CCL2/CCR2 signaling, is known to play a role in regulating the recruitment and polarization of macrophages during inflammation.^65^ Likewise, CXCL1 and CXCL12 are chemokines associated with chemotaxis and inflammation,^65^, ^66^ whereas both IL‐6 and IL‐8 are proinflammatory factors. Previous studies reported that IL‐6 induces the generation of collagen type I and plays a role in the response to infection and tissue injury.^67^, ^68^ Moreover, IL‐8, one of the key mediators of the inflammatory response, has been reported to have an important role in angiogenesis,^69^, ^70^ whereas SERPINE1 has been reported to play an important role in suppressing the adhesion, proliferation, and motility of endothelial and vascular smooth muscle cells.^71^, ^72^ Increased secretion of SERPINE1 has been observed during inflammation, physical injury, and exposure to angiotensin II. Further, SERPINE1 reportedly participates in the tissue injury repair program by inhibiting proliferation while promoting the migration of cells.^73^ As such, the decreased release of these cytokines from the hASC‐PDMS and hASC‐IA‐PDMS surfaces might further assist in reducing inflammation and increasing cell proliferation.

### 2.6 In vivo experiments

Here, hASC‐PDMS, IA‐PDMS, and hASC‐IA‐PDMS groups were established as treatment groups and PDMS was established as a control group, and hASC‐coated PDMS groups were referred to hASC‐PDMS and hASC‐IA‐PDMS groups. Categorized results of these studies are as follows.

#### 2.6.1 mRNA gene expression

**FIGURE 4:**
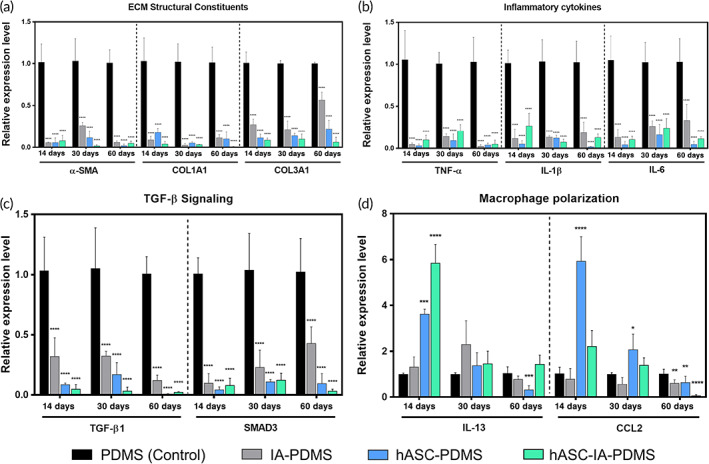
Quantitative real‐time PCR analysis for the expression of various genes on the surface of poly(dimethylsiloxane) (PDMS), itaconic acid (IA)‐PDMS, human adipose‐derived stem cell (hASC)‐PDMS, and hASC‐IA‐PDMS relative to the mRNA level of glyceraldehyde 3‐phosphate dehydrogenase (GAPDH) at 14, 30, and 60 days after implantation. Data are shown as the mean ± SD (*n* = 8). **p < 0.01, ***p < 0.001, and ****p < 0.0001 (one‐way ANOVA, Bonferroni)

We extracted mRNA from the capsule tissue and compared the expression levels of genes related to host reactions to foreign bodies (Table S1). Our analysis was divided into four categories, namely extracellular matrix (ECM) structural constituent genes (*α‐smooth muscle actin [SMA]*, *collagen 1 alpha 1 [COL1A1]*, and *collagen 3 alpha 1 [COL3A1]*), inflammation (*tumor necrosis factor [TNF]‐α*, *interleukin [IL]‐1β*, and *IL‐6*), transforming growth factor [TGF]‐β signaling (*TGF‐β1* and *SMAD3*), and M2 macrophage polarization‐related genes (*IL‐13* and *CCL2*) (Figure 4). The thickness of the capsule was determined based on the amount of accumulated collagen. When comparing the expression level of genes between groups, at each time point, we observed a significant decrease in the treatment groups compared with levels in the control group. In addition, we found that the expression of *α‐SMA* was significantly decreased in the treatment groups (*p* < 0.0001). When we investigated the expression of *TNF‐α*, *IL‐1β*, and *IL‐6* in the treatment groups at 14, 30, and 60 days, we discovered that the inflammatory cytokine‐related genes were expressed at a significantly lower level compared with those in the control group (*p* < 0.0001). The TGF‐β cytokine is known to be predominantly involved in fibrosis and affects the differentiation of fibroblasts into myofibroblasts.^74^ Additionally, it has been reported that TGF‐β‐induced synthesis of α‐SMA requires SMAD3. To confirm this, we compared the expression level of each of these factors and observed significantly lower expression levels of *TGF‐β1* and *SMAD3* at 14, 30, and 60 days, with a marginally lower average expression level, especially in the hASC‐coated groups (*p* < 0.0001). Finally, we investigated *CCL2* and *IL‐13*, which are known to facilitate the differentiation of macrophages toward an M2 phenotype.^57^ We observed a notable increase in the expression of *IL‐13* in the hASC‐coated groups in the initial 14 days, whereas a significantly increased expression level of *CCL2* was also shown in the hASC‐PDMS group at Day 14 (*p* < 0.0001).

#### 2.6.2 Inflammatory response around the implant

**FIGURE 5:**
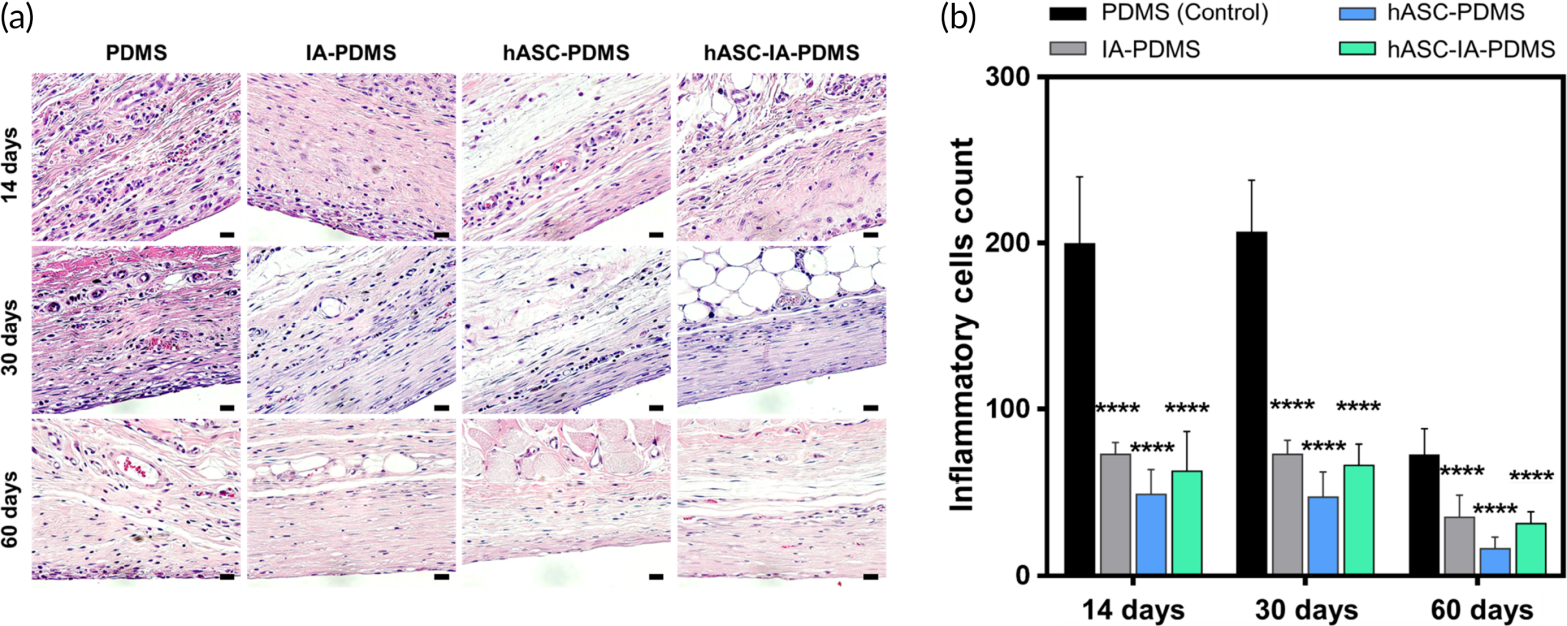
(a) Images of hematoxylin–eosin (H&E) staining of tissue slides and (b) evaluation of inflammatory cell count around implants after poly(dimethylsiloxane) (PDMS), itaconic acid (IA)‐PDMS, human adipose‐derived stem cell (hASC)‐PDMS, and hASC‐IA‐PDMS implantation at 14, 30, and 60 days. Each image was obtained using optical microscopy at 400× magnification and specifically analyzed for inflammatory mediated cells (i.e., neutrophils, basophils, and eosinophils; scale bars: 20 μm). Data are shown as the mean ± SD (*n* = 8). ****p < 0.0001 (one‐way ANOVA, Bonferroni)

An early stage of the cellular reaction during fibrosis development is the inflammation response, which involves several reactions comprising various inflammatory cells, such as eosinophils, neutrophils, and basophils. Inflammatory cells influence macrophage activities, result in fusion into foreign body giant cells, and finally create fibrosis.^45^ To evaluate the inflammation occurring around the implant, we stained the tissue with hematoxylin–eosin (H&E) and evaluated the result at 14, 30, and 60 days postimplantation. As shown in Figure 5, we initially noted severe inflammation in the control group; however, the degree of inflammation was significantly reduced in the treatment groups compared with that in the control group, from the onset (*p* < 0.0001). Among the treatment groups, the degree of inflammation was lowest in the hASC‐PDMS group; however, no significant difference was observed among the treatment groups at all time points (*p* < 0.05). Thus, we assumed that the surface in the treatment group was more biocompatible. The anti‐inflammatory effect observed was the strongest during the early stages, with the degree of inflammation in the control group decreasing to the level of inflammation in the initial treatment group at Day 60.

#### 2.6.3 Myofibroblasts

**FIGURE 6:**
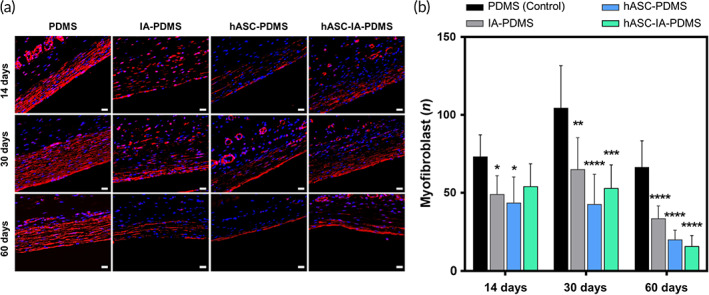
(a) Images of immunofluorescence staining of tissue slides and (b) evaluation of the number of myofibroblasts around implants after poly(dimethylsiloxane) (PDMS), itaconic acid (IA)‐PDMS, human adipose‐derived stem cell (hASC)‐PDMS, and hASC‐IA‐PDMS implantation at 14, 30, and 60 days. Each image was obtained using fluorescence microscopy at 400× magnification (scale bars: 20 μm). Data are shown as the mean ± SD (*n* = 8). *p < 0.05, **p < 0.01, ***p < 0.0001, and ****p < 0.0001 (one‐way ANOVA, Bonferroni)

The number of myofibroblasts is commonly used as an evaluation indicator for the determination of the degree of fibrosis,^75^ since more myofibroblasts present in the capsule will indicate greater contraction and therefore stronger pressure on the implant, resulting in the deformation of the implant and pain. As shown in Figure 6, myofibroblasts were primarily distributed in the region adjacent to the implant. Moreover, we noted a thicker layer of myofibroblasts in the control group than in the other groups, whereas the number of myofibroblasts in the treatment groups was significantly decreased compared with that in the control group (*p* < 0.05). This tendency was more noticeable at the 30‐ and 60‐day time points relative to that at 14 days.

#### 2.6.4 Macrophages

**FIGURE 7:**
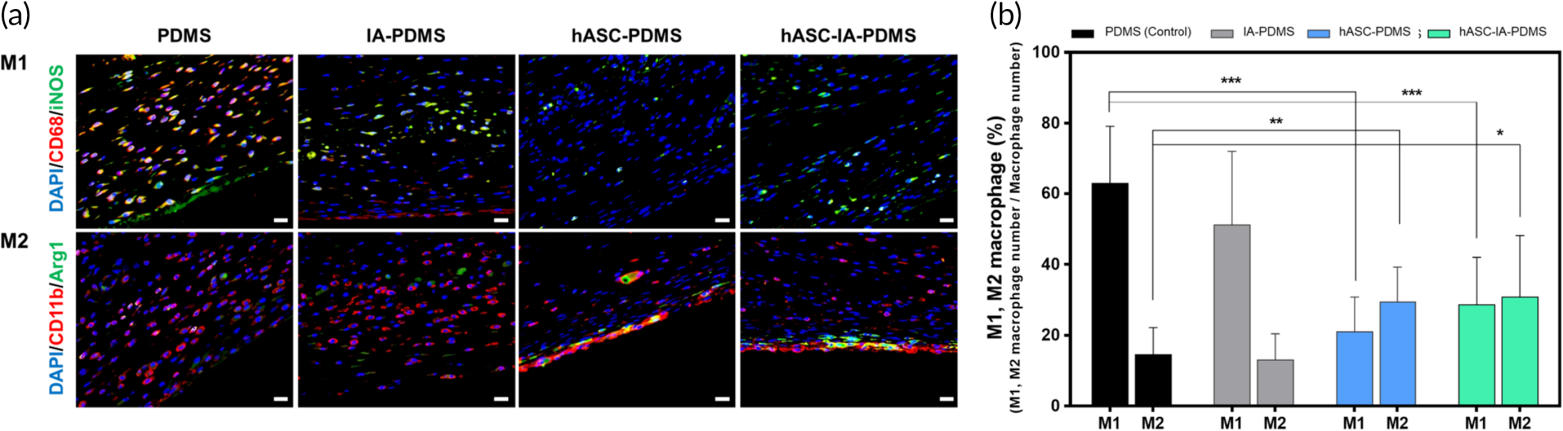
(a) Images of immunofluorescence staining of tissue slides and (b) evaluation of the percentage of M1 and M2 macrophages around implants after poly(dimethylsiloxane) (PDMS), itaconic acid (IA)‐PDMS, human adipose‐derived stem cell (hASC)‐PDMS, and hASCs‐IA‐PDMS implantation at 14 days. Representative percentages of M1 and M2 macrophages were calculated based on the number of M1 or M2 macrophages divided by the total number of macrophages. Each image was obtained using fluorescence microscopy at 400× magnification (scale bars: 20 μm). Data are shown as the mean ± SD (*n* = 8). *p < 0.05, **p < 0.01, and ***p < 0.0001 (one‐way ANOVA, Bonferroni)

Macrophages play an important role in the process of host reactions to foreign bodies. Monocytes can differentiate into different types of macrophages when exposed to different environments. For example, monocytes can differentiate into macrophages of the M1 (classical) and M2 (alternative) types; the M1 type primarily has a pro‐inflammatory role, whereas the M2 type has anti‐inflammatory and pro‐healing roles.^76^ We assumed that host macrophages would differentiate at different rates on each implant; thus, we analyzed their ratio by counting the number of macrophages of each type using the triple‐label immunofluorescence method. We used iNOS for M1 macrophages, whereas the M2 type was stained with arginase 1. Figure 7 shows the percentage of M1 and M2 macrophages around the implants. Upon analyzing the ratio of the M1/M2 macrophages on the slide at Day 14, we detected a higher ratio of M1 macrophages in the PDMS group. The IA‐PDMS group exhibited levels similar to those in the control group, but a lower percentage of M1‐type macrophages was observed. However, we observed a markedly lower number of M1 macrophages in the hASC‐coated groups, as well as a notable increase in the formation of M2 macrophages. This was possibly because hASCs coating affected the surrounding environment and thus the macrophage differentiation process. The presence of such an environment was evidenced by the expression level of *CCL2*/*IL‐13* in the capsule tissue (Figure 4).

#### 2.6.5 Capsule thickness and collagen density

**FIGURE 8:**
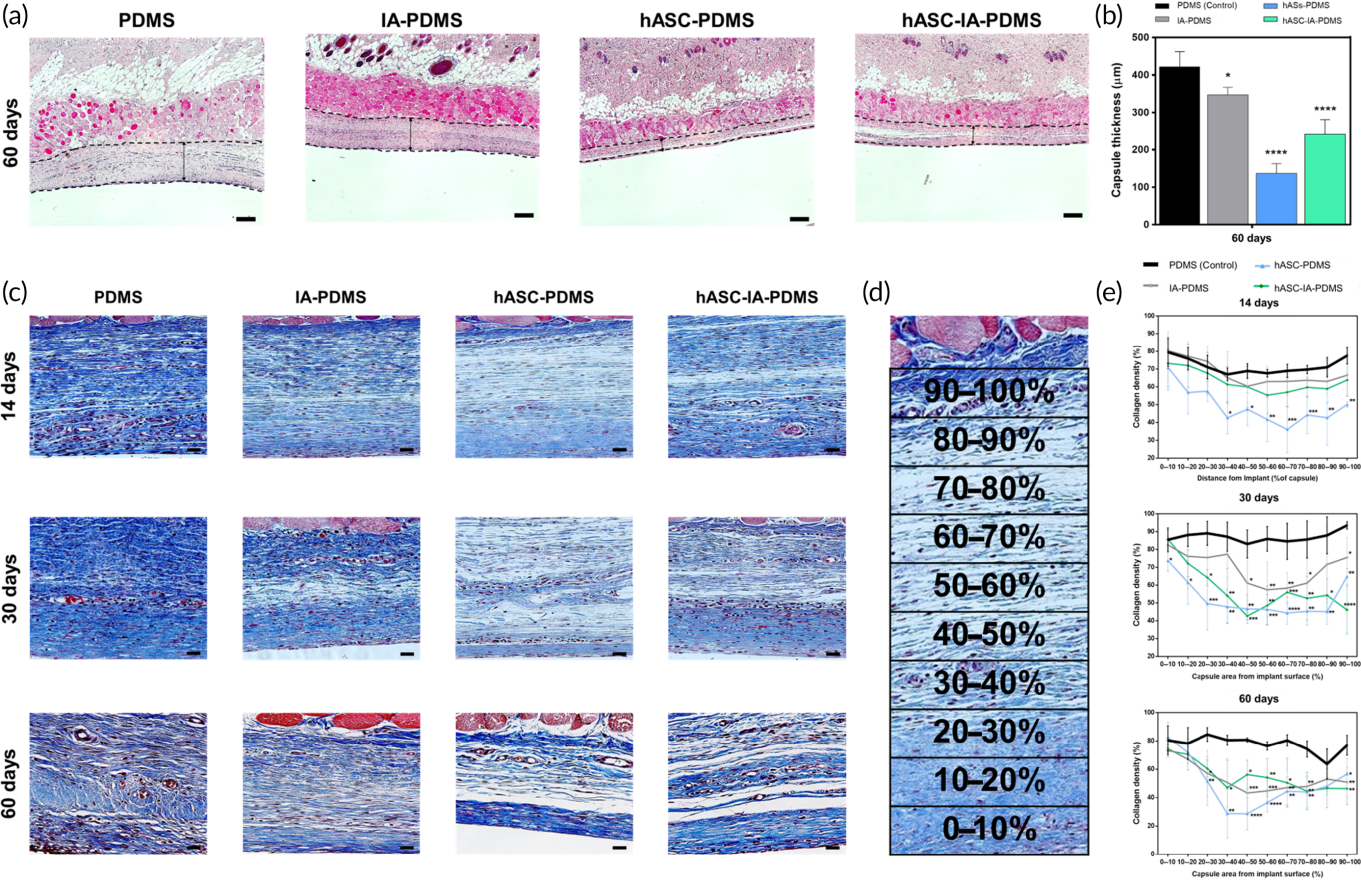
(a) Images of hematoxylin–eosin (H&E) staining of tissue slides and (b) evaluation of capsule thickness around implants after poly(dimethylsiloxane) (PDMS), itaconic acid (IA)‐PDMS, human adipose‐derived stem cell (hASC)‐PDMS, and hASCs‐IA‐PDMS implantation at 60 days. Each image was obtained using optical microscopy at 50× magnification. Double‐sided black dotted line indicates capsule thickness (scale bars: 200 μm). (c) Images of Masson's trichrome (MT) staining of tissue slides, (d) with data collected in the whole capsule area (100%) from the interface (at 10% increments), and (e) evaluation of the density of collagen deposition around implants. Collagen density was evaluated after PDMS, IA‐PDMS, hASC‐PDMS, and hASC‐IA‐PDMS implantation at 14, 30, and 60 days. Each image was obtained using optical microscopy with 400× magnification (scale bars: 20 μm). Data are shown as the mean ± SD (*n* = 4). *p < 0.05, **p < 0.01, ***p < 0.001, and ****p < 0.0001 (one‐way ANOVA, Bonferroni)

To verify the suppression of capsular contracture, we examined collagen density and capsule thickness. As shown in Figure 8a,b, a significant decrease was observed in capsule thickness in the IA‐PDMS group (*p* < 0.05) and the hASC‐coated groups (*p* < 0.0001) compared with that in the control group at day 60. More specifically, the capsule thickness was significantly reduced in the hASC‐coated groups compared with that in the IA‐PDMS group, such that the hASCs coating was considered to have the ability to suppress fibrosis (*p* < 0.0001). We also observed that the lowest capsule thickness occurred on the hASC‐PDMS surface and quantified the collagen density (Figure 8c,d) at each time point by determining the percentage of blue‐pixel coverage in the images within 100% (at 10% intervals) of the implant–tissue interface.^77^ At 14 days, in the dense capsule layer surrounding hASC‐PDMS, the collagen density close to the implant interface was >90% and was shown to decrease to approximately to 40% in the subcutaneous tissue next to the capsule (Figure 8c,d). The control group exhibited a uniform and high density on average, from the face of the implant to the muscle (0–100%). In the treatment groups, we observed lower collagen density in the middle region of the capsule. In particular, the hASC‐PDMS group showed significantly lower collagen density from the initial 14 days (*p* < 0.05); the other IA‐PDMS and hASC‐IA‐PDMS groups also showed a statistically significant decrease in density at 30 and 60 days (*p* < 0.05). Additionally, it was noted that the capsule of the hASC‐PDMS group was thinner than that of the hASC‐IA‐PDMS group (Figure 8a). Nevertheless, qualitative and semi‐qualitative determination of capsule thickness results could not completely refer to the degree of capsular contracture. Further, the hASC‐IA‐PDMS group showed superior quantitative results in the suppression of fibrosis‐related gene expression in vivo (Figure 4).

We hence considered that the reduction in collagen, which is the final fibrosis product, had resulted from an inflammatory cascade that began from the initial inhibition. We assumed that the in vivo host reaction to foreign bodies was inhibited via the recognition of an environment similar to the ECM due to hASCs coating. This biocompatible surface influenced differentiation into M2, and not M1, macrophages involved in the inflammatory reaction. Thus, the healing process appeared to have started early, reducing the infiltration of immune‐related cells, and resulting in the initiation of a chain reaction to detect foreign bodies. The IA‐PDMS group also showed superior fibrotic suppression compared with the control group in terms of collagen density and qPCR results. However, in terms of the final cumulative amount of collagen, the capsule thickness was associated with a more pronounced difference in the hASC‐coated group, possibly due to the difference in the differentiation ratio of M1/M2 macrophages (Figure 7). In addition, these differences might have resulted not only from the biocompatibility of the implant surface but also from the effect of the cytokines secreted by the cells attached to it. Thus, we found that not only hASC‐IA‐PDMS was effective in reducing the formation of capsular contracture, as was hypothesized, but that hASC‐PDMS also exhibited comparable anti‐fibrotic ability. Further studies on the effect of cell‐to‐cell interactions on fibrotic reactions in these microenvironments are warranted.

## 3 METHODS

### 3.1 Preparation of human adipose‐derived stem cells coated poly(dimethylsiloxane)

PDMS and IA‐PDMS were prepared as described in the Supporting Information. PDMS and IA‐PDMS samples were sterilized at 38°C for 4 h using an ethylene oxide sterilizer (SE30, ALOPS Corp., Gunpo, Korea). hASCs were cultured using Dulbecco's modified Eagle's medium low‐glucose (HyClone Laboratories, Logan, UT) containing 10% fetal bovine serum (HyClone) and 1% antibiotic/antimycotic solution (HyClone). The culture media were changed every 2 days. Trypsin (0.25%; 1×) solution (HyClone) was used to harvest cultured cells. hASCs (2 × 10^4^ cells) were seeded on PDMS and IA‐PDMS surfaces and incubated in an incubator (HERAEUS BB 15, Thermo Fisher Scientific, Seoul, Korea) at 37°C with 5% CO_2_. Samples with cells at 80% confluence (Day 3) were selected for use in in vivo experiments (Figure 1b). Isolation and characterization methods of hASCs are described in the Supporting Information. The use of hASCs was approved by the Chung‐Ang University Hospital Institutional Review Board and conducted as specified by the guidelines of the Declaration of Helsinki (IRB No. 2151‐005‐463).

### 3.2 Surface characterization of PDMS and IA‐PDMS

To observe the surface morphologies of hASCs on plates, PDMS, IA‐PDMS, hASC‐PDMS, and hASC‐IA‐PDMS, we used SEM (S‐3400 N; Hitachi, Tokyo, Japan) using a 10 kV acceleration voltage after coating with platinum layers via ion sputtering (E‐1010; Hitachi) for 120 s. For samples with hASCs on the surface (hASC‐PDMS and hASC‐IA‐PDMS), the appropriate number of cells at 80% confluence was used. The IA‐PDMS was characterized using a water contact angle analyzer (Phoenix‐MT, Surface Electro Optics, Suwon, Korea) and ATR/FTIR spectroscopy (Vertex 70, Bruker, MA). The surface stability of IA‐PDMS was examined based on the water contact angel, ATR‐FTIR, in vitro protein adsorption, in vitro anti‐bacterial adhesion, and in vitro cell studies at various time intervals over 60 days, as described in the Supporting Information.

### 3.3 In vitro cell cytotoxicity and characterization of cell morphology

PDMS or IA‐PDMS samples with cultured cells were washed twice with DPBS (modified 1×, pH 7.4, HyClone) before determining in vitro cell cytotoxicity activities. The cell viability and morphology of hASCs cultured on PDMS or IA‐PDMS surfaces were examined, respectively, using the LIVE/DEAD Viability/Cytotoxicity Kit for mammalian cells (Thermo Fisher, Waltham, MA) and rhodamine‐phalloidin/DAPI staining at 1, 3, and 7 days. Cell adhesion and proliferation at 1, 3, and 7 days were determined using the CCK‐8 assay. We also performed the MTT assay at 1, 3, and 7 days to evaluate cytocompatibility. All cell study details methods are described in the Supporting Information.

### 3.4 Cytokine release

hASCs (2 × 10^4^ cells) were cultured on culture plates (control), PDMS, and IA‐PDMS in culture medium without FBS, and no media changes were performed. On Day 3, the collected supernatant was added to the Proteome Profiler™ Human Cytokine Array Kit (R&D Systems Inc., Minneapolis, MN). The cytokine assay was conducted according to the manufacturer's protocol. Chemiluminescence (ChemiDoc, Bio‐Rad, CA) was used for the visualization and evaluation of protein quantities.

### 3.5 In vivo experiments

For in vivo evaluation, 48 9‐week‐old Sprague Dawley rats (200–250 g) were used. During animal experiments, all animals were housed in a specific pathogen‐free room under a 12 h day/night cycle, with free access to water and food. All experiments and the methodology used in this research were approved by the IACUC of Seoul National University Bundang Hospital (approval number: BA1903‐268/015‐01). To examine the antifibrotic ability of the surface‐modified implants, the dorsal region of rats was completely shaved of any hair. Rats were then randomly divided into four groups of four rats each. Subsequently, incisions (2 cm in length) were made using a pair of surgical scissors. A subcutaneous pocket was made through the incision area, and the sample was inserted into the pocket. PDMS was inserted in Group 1 (control group), IA‐PDMS was inserted in Group 2, hASC‐PDMS was inserted in Group 3, and hASC‐IA‐PDMS was inserted in Group 4. In each rat, two pieces of samples were inserted parallel to the dorsal side. No DPBS washing was performed for hASC‐PDMS and hASC‐IA‐PDMS. The incision was subsequently sutured using Nylon 4/0 (ETHILON, New Brunswick, NJ) and disinfected with betadine solution to prevent external stimulation and infection. At 14, 30, and 60 days, CO_2_ euthanasia was performed for biopsy and tissue analysis. Accordingly, these tissues were collected for quantitative analysis and in vivo staining analysis. For quantitative analysis, tissues were put in a cryovial, immediately placed in liquid nitrogen, and stored at −80°C until use. For in vivo staining analysis, tissues were stored in 4% formalin for 1 day. A paraffin block was subsequently prepared using biopsy tissue. Slices (thickness, 4 μm) were made and stained for each factor before tissue staining.

#### 3.5.1 Real‐time quantitative polymerase chain reaction

Total RNA was extracted from biopsy tissue using Trizol reagent (Invitrogen, Carlsbad, CA) according to the manufacturer's instructions. Then, RNA (1 μg) was used to synthesize cDNA using the AccuPower® RocketScript™ RT‐PCR PreMix & Master Mix (Bioneer, Daejeon, Korea). The synthesized cDNA was stored at −20°C until use. Real‐time quantitative polymerase chain reaction (RT‐qPCR) analysis was performed using the Power SYBR Green PCR Master Mix (Thermo Fisher) and data analysis was performed using a StepOnePlus Real‐Time PCR system (AB Applied, Life Technologies, MA). Table S2 shows the primer sequences for the glyceraldehyde 3‐phosphate dehydrogenase (*GAPDH*) reference gene and target genes. The expression level of each target mRNA was normalized to that of *GAPDH* and compared with that of the control group.

#### 3.5.2 Histological analysis

To confirm the efficacy of the antifibrotic functional implants, we performed histological analysis of the capsule thickness and collagen density and counts of inflammatory cells and myofibroblasts. Capsule thickness and inflammatory responses were analyzed using the H&E stain kit (H‐3502; Vector Laboratories, Burlingame, CA), collagen density was analyzed using a Masson's Trichrome (MT) stain kit (Sigma‐Aldrich), and myofibroblasts, a fibrotic‐related factor, were analyzed using immunofluorescence staining. For capsule thickness analysis, we used H&E‐stained slides to obtain 50× optical microscopy tissue images, whereas capsule thickness was analyzed using at least four images per group. Capsules with the least thickness from the H&E images were selectively analyzed. For collagen density analysis, tissue images were acquired using 400× optical microscopy of MT‐stained slides. The blue bundle was specifically selected, and the selected area was changed into a % value relative to the total image area. All % values were objectively analyzed using ImageJ software (ver. 1.47, National Institutes of Health, Rockville, MD) in a blinded manner. Additionally, to verify the antifibrotic effect, the number of myofibroblasts was quantitatively evaluated and analyzed by immunofluorescence staining using an anti‐vimentin rabbit antibody (ab92547; Abcam, Cambridge, MA) and anti‐alpha smooth muscle actin mouse antibody (ab7817; Abcam) for selectivity. Each antibody was diluted at a ratio of 1:200 (vimentin) and 1:100 (alpha‐smooth muscle actin), and 1:2000‐diluted secondary antibodies (Alexa Fluor 488 rabbit anti‐mouse IgG [H + L] [A11059; Thermo Fisher] and Alexa Fluor 488 goat anti‐mouse IgG [H + L] [A11001; Thermo Fisher]) were used for the detection of fluorescence signals with a 1:2000 diluent solution. Analysis was performed using fluorescence microscopy, and samples were selectively analyzed based on 488 nm fluorescence signals emitted from specifically bound antibodies.

### 3.6 Quantification and statistical analysis

Statistical analysis was performed using GraphPad Prism 7 (GraphPad Software, San Diego, CA). One‐way ANOVA followed by Bonferroni's multiple comparison tests was performed. Technical replication was performed in triplicate for each analysis, and total *n* and SD values are shown in the figure legends. An alpha value of 0.05 was used for all statistical analyses.

## 4 CONCLUSION

Herein, we attempted the surface modification of PDMS using hASCs, as well as IA, to minimize the host reactions to foreign bodies, which appear as a side effect in the use of several medical devices. Both the in vitro and in vivo studies demonstrated that fibrosis was further suppressed on the hydrophilic surface modified with IA and hASCs compared to that with the hydrophobic bare PDMS surface. In addition, we confirmed that macrophage differentiation occurred toward the M2 phenotype in the group in which the surface of PDMS was modified using hASCs, further enhancing the antifibrotic ability in an anti‐inflammatory environment. Overall, the fibrotic effect was lowest in hASC‐PDMS and hASC‐IA‐PDMS, based on the following factors: capsule thickness, collagen density, and number of myofibroblasts and fibroblasts. This suggested that this cell coating had outstanding antifibrotic ability. Through these results, we have provided insights into future surface modification approaches to reduce the capsular contraction of medical devices. In future work, investigating clinical oncology might prove important.

## AUTHOR CONTRIBUTIONS

**Chanutchamon Sutthiwanjampa:** Data curation (lead); formal analysis (lead); investigation (lead); methodology (lead); visualization (lead); writing – original draft (lead); writing – review and editing (lead). **Byung Ho Shin:** Data curation (equal); formal analysis (equal); investigation (equal); methodology (lead); writing – original draft (lead); writing – review and editing (equal). **Na Eun Ryu:** Formal analysis (equal); methodology (equal). **Shin Hyuk Kang:** Project administration (equal); supervision (supporting); validation (supporting). **Chan Yeong Heo:** Conceptualization (equal); funding acquisition (lead); project administration (lead); supervision (equal); validation (lead); writing – review and editing (supporting). **Hansoo Park:** Conceptualization (lead); funding acquisition (lead); project administration (lead); supervision (lead); validation (lead); writing – review and editing (equal).

## CONFLICT OF INTERESTS

The authors declare no conflict of interest.

### PEER REVIEW

The peer review history for this article is available at https://publons.com/publon/10.1002/btm2.10260.

## Supporting information


**Appendix** **S1**: Supporting Information

## Data Availability

The data that support the findings of this study are available from the corresponding author upon reasonable request.
