# Supplementary material for: Assessment of human adipose‐derived stem cell on surface‐modified silicone implant to reduce capsular contracture formation
Source: Bioeng Transl Med. 2021 Oct 8;7(1):e10260. doi: 10.1002/btm2.10260 (PMC8780897; doi:10.1002/btm2.10260)
Supplement: Supplementary file 1 — Appendix S1: Supporting Information [file BTM2-7-e10260-s001.docx]

SUPPORTING INFORMATION

Assessment of Human Adipose-Derived Stem Cell on Surface-Modified Silicone Implant to Reduce Capsular Contracture Formation

Chanutchamon Sutthiwanjampa^ǂ^, Byung Ho Shin^ǂ^, Na Eun Ryu, Shin Hyuk Kang, Chan Yeong Heo*, and Hansoo Park*

**SUPPLEMENTAL METHODS**

**Preparation of Poly(dimethylsiloxane) Membrane**

To prepare the PDMS membrane, we first mixed 1 g of curing agent and 10 g of base of the Sylgard® 184 Silicone Elastomer Kit (Dow Corning, Midland, MI). The mixture was then spread on a 100-mm petri dish, which was subsequently subjected to degasification using a vacuum desiccator until no an air bubble was observed. Then, the degassed mixture was placed in an oven at 60 °C for 60 min. The obtained PDMS membranes were punched into circular disks with a diameter of 1.2 cm **(Figure 1A)**.

**Preparation of Itaconic Acid-Conjugated Poly(dimethylsiloxane)**

IA-PDMS samples were prepared as previously described.^1^ Briefly, cleaned PDMS samples were treated with fixed O_2_ flow at 20 sccm and a power supply of 100 W at a pressure of 5 × 10^-2^ torr for 1 min (CUTE-1B, Femto Science, Hwaseong, Korea). The O_2_-treated samples were subsequently incubated in 5 wt % (3-aminopropyl)triethoxysilane (APTES; Sigma-Aldrich, St. Louis, MO) in distilled water (DW) at 200 rpm and 60 °C for 120 min. APTES-treated samples were washed twice with DW to remove any unreacted APTES molecules. IA solutions (150 mM; Sigma-Aldrich) were prepared in DW with 50 mM 1-ethyl-3-(3-dimethylaminopropyl)carbodiimide (EDC; Sigma-Aldrich) and 50 mM *N*-hydroxysuccinimide (NSH; Sigma-Aldrich). APTES-treated samples were incubated in an IA reaction mixture at 200 rpm and 60 °C for 120 min. Unreacted IA, EDC, and NHS residues were washed away with DW.

**Contact Angle Measurement**

The hydrophilicity of the bare PDMS and IA-PDMS surfaces was measured using a contact angle analyzer (Phoenix‐MT, Surface Electro Optics, Suwon, Korea).

**Attenuated Total Reflectance – Frontier Transform Infrared Spectroscopy**

To characterize a chemical bond on the bare PDMS and IA-PDMS surface, an Attenuated total reflectance – Frontier transform infrared spectroscopy (ATR-FTIR; Nicolet 6700, Thermo Scientific, USA) was used with an average of 25 scans, and a resolution of 4 cm^−1^ at 25 °C. The spectrum was recorded at a measurement raged from 400 to 4000 cm^−1^.

***In Vitro* Protein Adsorption Assay**

The bare PDMS and IA-PDMS were separately equilibrated by incubating in 1× PBS (HyClone, Logan, UT) at 300 rpm and 25 °C overnight. The equilibrated samples were incubated with 4.5 mg/mL bovine serum albumin (BSA; Sigma-Aldrich) in PBS at 200 rpm and 37 °C for 120 min. After washing twice with PBS, the BSA-incubated samples were transferred into a 24-well plate. Sodium dodecyl sulfate (1 % w/w in PBS) 1 mL was added to each well, following sonication for 20 min. Then, the supernatant (200 μL) was transferred into a new 24-well plate containing 600 μL of the working reagent mixture (MA:MB:MC at 25:24:1 (v/v/v); Micro BCA™ Protein Assay Kit, Thermo Fisher Scientific, Waltham, MA). The plate was incubated at 200 rpm and 37 °C for 120 min in the dark before absorbance measurement in a microplate spectrophotometer at 570 nm.

**Bacterial Anti-Adhesion and Biofilm Formation**

*Staphylococcus epidermidis* (ATCC 35984; Manassas, VA), *Staphylococcus aureus* (ATCC 35556), *Pseudomonas aeruginosa* (ATCC 27853), and *Ralstonia pickettii* (ATCC 27511) were selected for evaluation of the bacterial adhesion property on bare PDMS and IA-PDMS as they are bacterial species commonly related to bacterial isolated from implants causing capsular contracture.^2, 3^ Samples were sterilized with ethylene oxide gas. Bacterial cell suspensions were separately prepared in Difco™ nutrient medium (BD bioscience, Franklin Lakes, NJ). Each sample was then immersed in 2 mL of bacterial suspension and incubated in a shaking incubator at 60 rpm and 37 °C. After 8 h of incubation, the samples were washed twice with Dulbecco’s phosphate-buffered saline (DPBS; modified 1×, pH 7.4, HyClone), stained with LIVE/DEAD™ BacLight™ bacterial viability kit (1 mL of a SYTO 9 and propidium iodide mixture; Thermo Fisher Scientific) according to manufacturer’s instructions for 15 min in the dark, and observed by fluorescence microscopy.

**Isolation of Human Adipose-Derived Stem Cell**

hASCs were isolated from informed healthy, non-inflammation or cancer female patients who underwent liposuction. Briefly, the obtained lipoaspirate was washed twice with DPBS containing 1 % antibiotic/antimycotic solution. Enzymatic digestion was performed using 0.01 % collagenase type I (Sigma-Aldrich) in a humidified 5 % CO_2_ incubator for 60 min at 37 °C with occasional shaking. Cells were centrifuged at 1,300 rpm for 3 min resulting in stromal vascular fraction (SVF). The SVF was resuspended in a culture medium (DMEM supplemented with 10 % fetal bovine serum, and 1 % antibiotic/antimycotic), passed through a 100 µm cell strainer (BD Biosciences, Bedford, MA), transferred into cell culture disk, and incubated at 37 °C in a humidified 5 % CO_2_ incubator. hASCs at passages 3-6 were used in this study.

**Characterization of Human Adipose-Derived Stem Cell**

The hASCs were characterized by examination of an expression of surface markers including CD14, CD34, CD45, CD73, and CD105. The hASCs were harvested from a monolayer culture dish using trypsin and prepared as single-cell suspension, and stained by specific antibodies including FITC-conjugated CD14, CD45, and PE-conjugated CD34, CD73 (BD Pharmingen, San Jose, CA). Flow cytometric analysis of the samples was conducted using BD FACS AriaII (Becton Dickinson, NJ). Data were analyzed using Flowjo software (Treestar, Woodburn, OR).

***In Vitro* Cell Cytotoxicity and Characterization of Cell Morphology**

PDMS or IA-PDMS samples with cultured cells were washed twice with DPBS before determining *in vitro* cell cytotoxicity activities. The cell viability of hASCs cultured on PDMS or IA-PDMS surfaces was examined using the LIVE/DEAD Viability/Cytotoxicity Kit for mammalian cells (Thermo Fisher, Waltham, MA) at 1, 3, and 7 days. Cells were stained according to the manufacturer’s procedure and incubated at 37°C and 5 % CO_2_ for 30 min. To analyze cell morphology, after washing with DPBS, cells were fixed with 4 % paraformaldehyde (FUJIFILM Wako Pure Chemical, Osaka, Japan) for 20 min followed by permeabilization with 1 % Triton X-100 (Sigma-Aldrich) for 10 min. The cells were then stained with rhodamine-phalloidin (1 mL; Thermo Fisher) for 30 min after which the solution was removed from each well before the subsequent staining with DAPI (1 mL; Thermo Fisher) for 3 min. Stained cells were examined under fluorescence microscopy using a model OX.2053-PLPH (Euromax, Roermond, Netherlands).

Cell adhesion and proliferation at 1, 3, and 7 days were determined using the CCK-8 assay. Briefly, 10 % CCK-8 solution (Dojindo, Kumamoto, Japan) in DMEM (1 mL) was added to each well in a 24-well plate containing cell-seeded samples and incubated at 37 °C and 5 % CO_2_ for 120 min. A microplate spectrophotometer (Biotek Instruments, VT) with a fixed wavelength of 450 nm was used to measure the absorbance. The linear regression equation of the standard curve was calculated according to the number of cells and the corresponding OD value and used to determine the number of viable cells. We also performed the MTT assay at 1, 3, and 7 days to evaluate cytocompatibility. Following cell seeding and adhesion on the PDMS and IA-PDMS surfaces, samples were transferred to new plates to exclude cells on the plate. Thereafter, 10 % v/v MTT (Sigma-Aldrich) in the growth medium was added to cell-seeded samples, incubated at 37 °C and 5 % CO_2_ for 60 min and dissolved in dimethyl sulfoxide (Sigma-Aldrich) to obtain purple formazan. After transferring the solution (200 μL) to 96-well plates, the absorbance was measured at 540 nm using a microplate spectrophotometer.

**Stability of Itaconic Acid-Conjugated Poly(dimethylsiloxane)**

After conjugation of IA on the PDMS surface, the samples were either left exposed to air or kept in a petri dish with de-ionized (DI) water or DPBS. Measurement of water contact angle was performed at days 0, 1, 3, 7, 14, 30, and 60 after storage. Examination of ATR-FTIR, amount of absorbed protein, cell studies (cells adhesion patter, cell morphology, cell cytotoxicity), and bacterial adhesion was carried out at days 0, 30, and 60 after storage.

**SUPPLEMENTAL FIGURES**


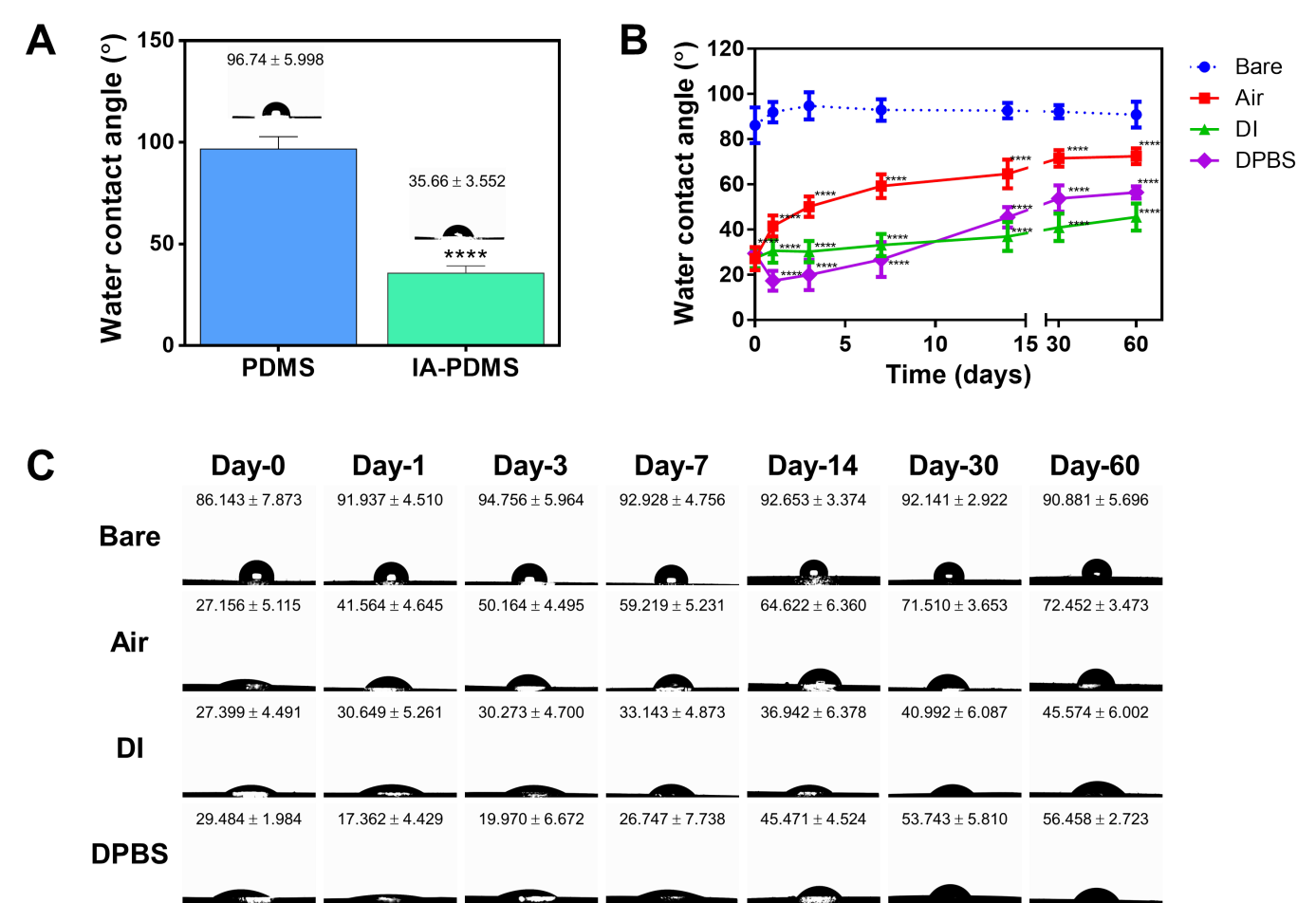


**Figure S1.** **(A)** Water contact angle on poly(dimethylsiloxane) (PDMS) and itaconic acid (IA)-PDMS surfaces. Inserted images show the contact angle analysis for each sample. **(B)** Water contact angle values and **(C)** images of poly(dimethylsiloxane) (PDMS) and itaconic acid (IA)-PDMS surfaces at various time points under air-exposure, deionized-water (DI) storage, and Dulbecco’s Phosphate Buffered Saline (DPBS) storage conditions. Embedded numbers show the contact angle values (°). Data are shown as the mean ± SD (n = 10). *****p* < 0.0001 (one-way ANOVA, Bonferroni). IA-PDMS maintained hydrophilicity on the PDMS surface with water contact values of 72.452 ± 3.743° significantly lower than 90.881 ± 5.696° of the bare PDMS when exposed to the air for 60 days (*p* < 0.0001).


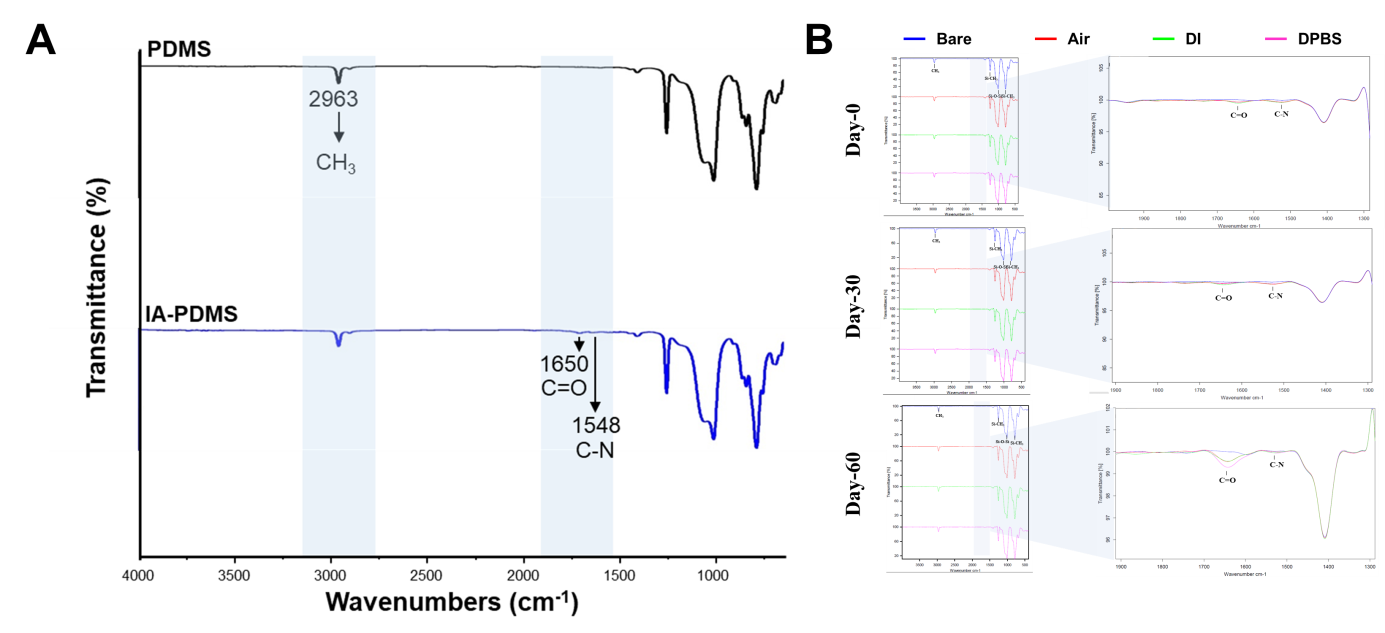


**Figure S2. (A)** Attenuated total reflectance/Fourier transform infrared (ATR-FTIR) spectra of poly(dimethylsiloxane) (PDMS) and itaconic acid (IA)-PDMS surfaces. **(B)** ATR-FTIR spectra showed the peaks of C=O and C-N groups, indicating the formation of IA on the PDMS surface under air-exposure, deionized-water (DI) storage, and Dulbecco’s Phosphate Buffered Saline (DPBS) storage conditions for up to 60 days.


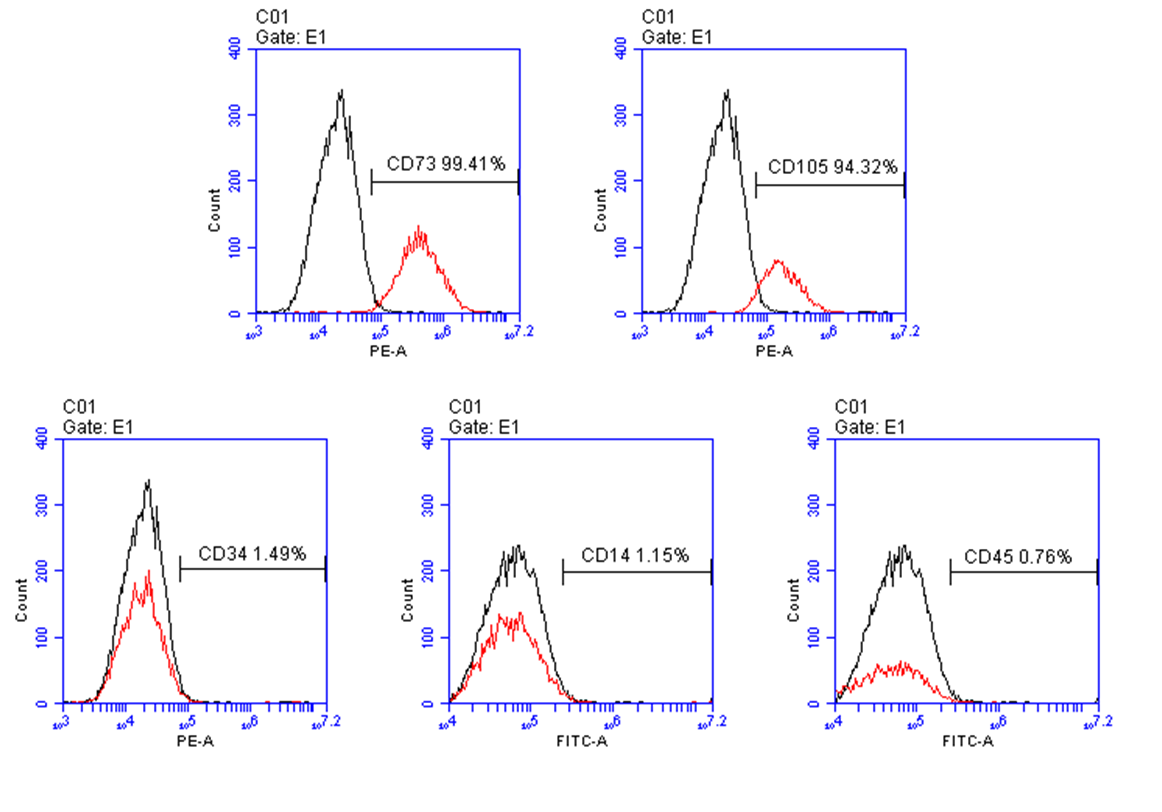


**Figure S3.** Expression of cell surface CD markers of hASCs from lipoaspirate cultured *in vitro* at passage 4 after being plated in tissue culture disks for 4 days using flow cytometry analysis. The isolated hASCs expressed high levels of CD73 and CD105 and low levels of CD34, CD14, and CD45.

**Figure S4.** *In vitro* protein adsorption on poly(dimethylsiloxane) (PDMS) and itaconic acid (IA)-PDMS surfaces at various time points under air-exposure, deionized-water (DI) storage, and Dulbecco’s Phosphate Buffered Saline (DPBS) storage conditions. On day 0, after IA conjugation, the average amount of BSA adsorbed on PDMS, air-exposed IA-PDMS, IA-PDMS in DI storage, and IA-PDMS in DPBS storage were 0.023500 ± 0.002369, 0.000649 ± 0.000943, 0.000386 ± 0.000468, and 0.00719 ± 0.001439 mg/cm^2^, respectively. At day-30, after IA conjugation, the average amount of BSA adsorbed on PDMS, air-exporsured IA-PDMS, IA-PDMS in DI storage, and IA-PDMS in DPBS storage were 0.022877 ± 0.003459, 0.008447 ± 0.001929, 0.001149 ± 0.001022, and 0.005553 ± 0.000876 mg/cm^2^, respectively. At day 60, after IA conjugation, the average amount of BSA adsorbed on PDMS, air-exposed IA-PDMS, IA-PDMS in DI storage, and IA-PDMS in DPBS storage were 0.023807 ± 0.003093, 0.017833 ± 0.002146, 0.006284 ± 0.000464, and 0.008535 ± 0.001085 mg/cm^2^, respectively. At every time point, IA-PDMS in all of the storage conditions (air, DI, and DPBS) showed surface protein absorption significantly lower than those of the bare PDMS (*p* < 0.0001). Data are shown as the mean ± SD (n = 4). *****p* < 0.0001 (one-way ANOVA, Bonferroni).


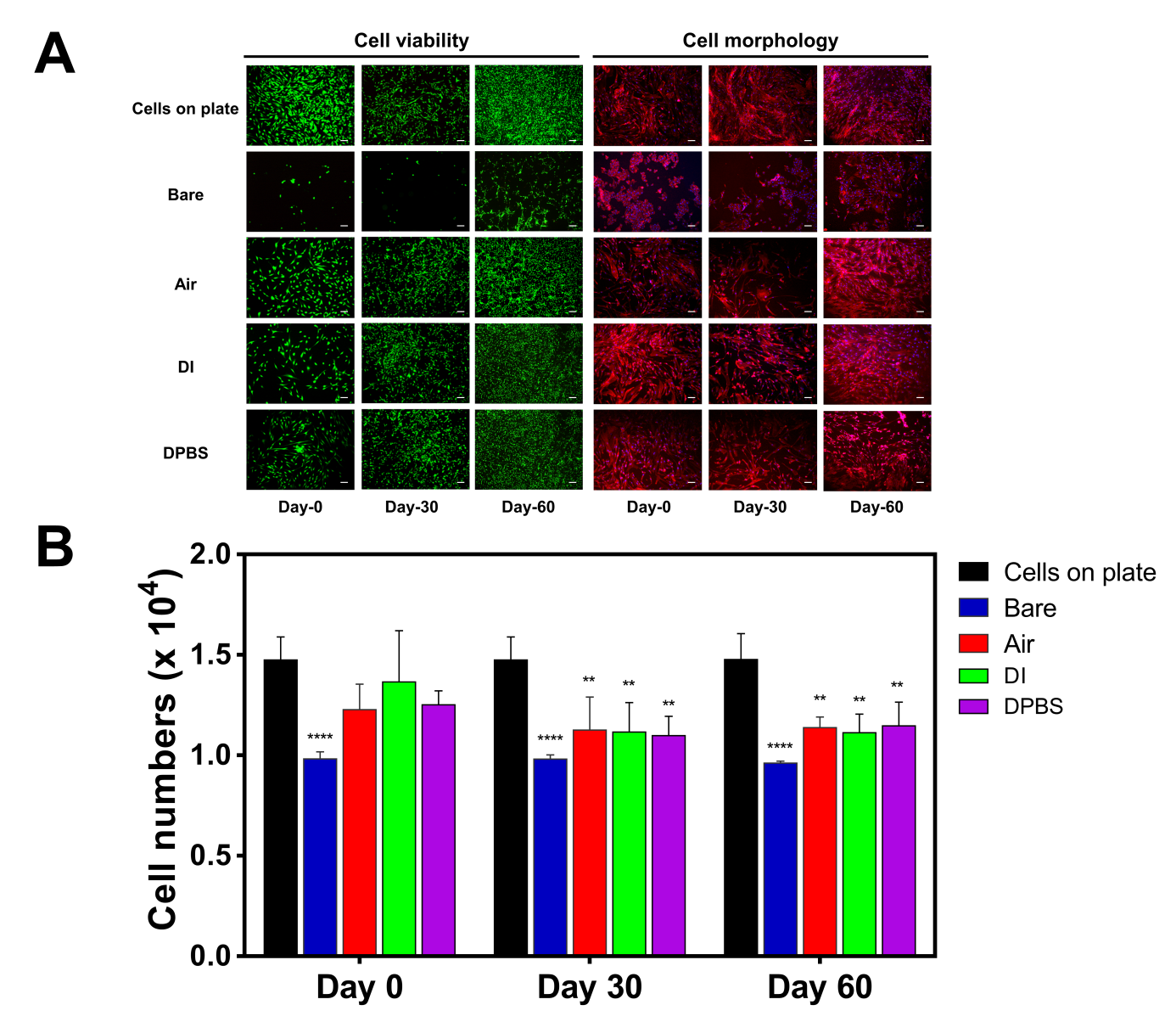


**Figure S5. (A)** Fluorescence micrograph of *in vitro* cell viability and **(B)** cell morphology of human adipose-derived stem cells (hASCs) at day 7 on a culture plate, hASC-poly(dimethylsiloxane) (PDMS), and hASC-itaconic acid (IA)-PDMS at various storage time points under air-exposure, deionized-water (DI) storage, and Dulbecco’s Phosphate Buffered Saline (DPBS) storage conditions at a magnification of 4× (for cell viability) or 10× (for cell morphology), and results of **(C)** cell proliferation from the CCK-8 assay for hASCs cultured for 7 days on a culture plate, PDMS, and IA-PDMS at various storage time points under air-exposure, DI storage, and DPBS storage conditions (scale bars: 20 μm). At every storage time point under every storage condition, the cells were evenly distributed with long and widely spread morphology on the surfaces of IA-PDMS, exhibiting the same pattern as those on a culture plate. On the contrary, the cells were spherical and clustered on the PDMS surface. Cells numbers on PDMS were significantly lower than those on culture plates (control) at every storage time point (*p* < 0.0001), whereas those on IA-PDMS under every storage condition were significantly lower than those on culture plates at 30 and 60 days after storage (*p* < 0.01). However, although cell proliferation on IA-PDMS under every storage condition was significantly lower than that on the culture plate, they showed cell amounts higher than that on PDMS at every storage time point. (scale bars: 20 μm). Data are shown as the mean ± SD (n = 3). ***p* < 0.01, and *****p* < 0.0001 (one-way ANOVA, Bonferroni).


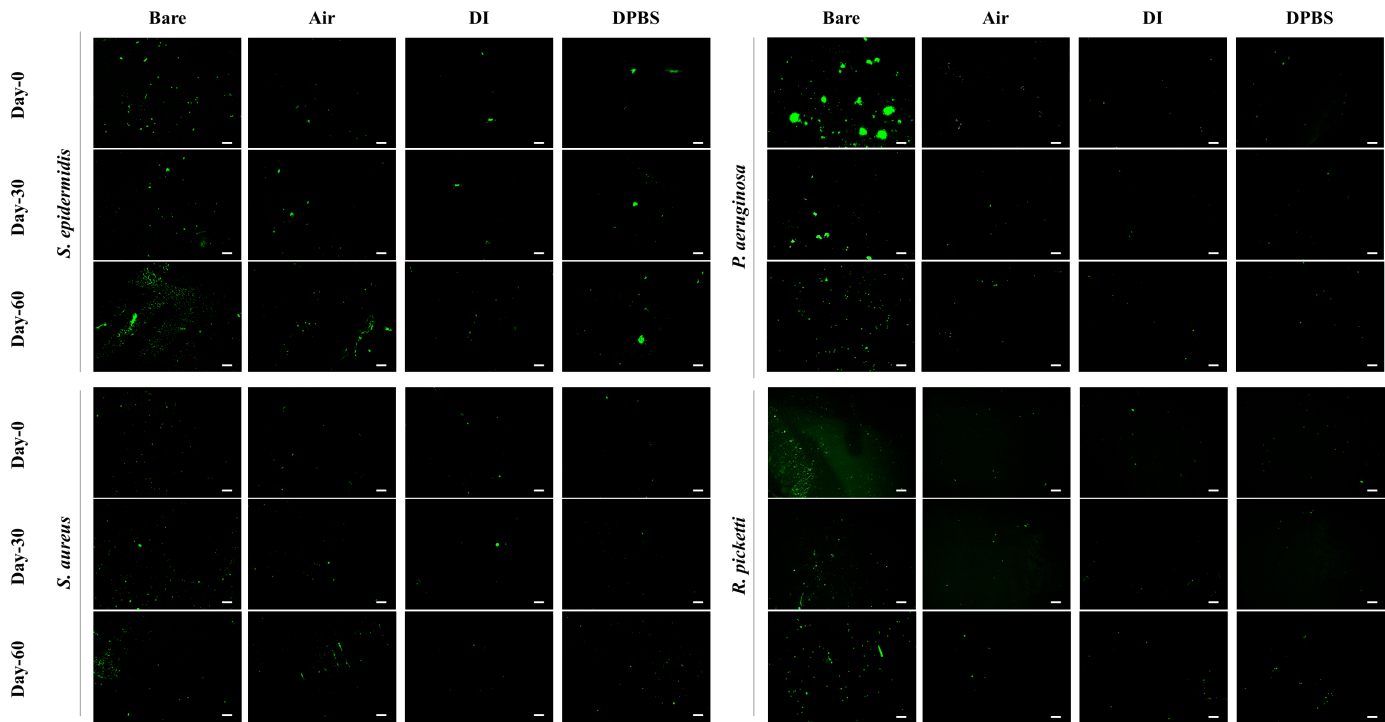


**Figure S6.** Fluorescence images of *in vitro* anti-bacterial adhesion effects against biofilm-forming bacterial including *S. epidermidis*, *S. aureus,* *P. aeruginosa*, and *R. Pickettii* on bare PDMS and IA-conjugated PDMS surface at day 0, day 30, and day 60 under air-exposure, deionized-water (DI) storage, and Dulbecco’s Phosphate Buffered Saline (DPBS) storage conditions. The images were observed at 4× magnification. A decrease in the adhesion of every bacterial species, especially *P. aeruginosa*, on the IA-PDMS surface was observed under every storage condition for up to 60 days.

**Table S1.** Relative cytokines mRNA expression in PDMS implants

| **Cytokines** | **14 days** | | | | **30 days** | | | | **60 days** | | | |
| --- | --- | --- | --- | --- | --- | --- | --- | --- | --- | --- | --- | --- |
|  | **Group 1**  **(Control)** | **Group 2** | **Group 3** | **Group 4** | **Group 1**  **(Control)** | **Group 2** | **Group 3** | **Group 4** | **Group 1**  **(Control)** | **Group 2** | **Group 3** | **Group 4** |
| **COL1A1** | 1.029 ± 0.2814 | 0.08796 ± 0.04566^****^ | 0.1786 ± 0.05013^****^ | 0.03865 ± 0.02954^****^ | 1.020 ± 0.2198 | 0.02466 ± 0.02005^****^ | 0.05189 ± 0.01582^****^ | 0.03212 ± 0.005469^****^ | 1.012 ± 0.1850 | 0.1146 ± 0.03789^****^ | 0.1013 ± 0.08292^****^ | 0.007189 ± 0.003915^****^ |
| **COL1A3** | 1.007 ± 0.1313 | 0.2718 ± 0.06536^****^ | 0.1150 ± 0.02895^****^ | 0.08658 ± 0.02446^****^ | 1.001 ± 0.03861 | 0.2140 ± 0.1027^****^ | 0.1394 ± 0.02672^****^ | 0.09822 ± 0.06070^****^ | 1.000 ± 0.01691 | 0.5654 ± 0.09596^****^ | 0.2198 ± 0.1062^****^ | 0.06198 ± 0.05775^****^ |
| **SMAD3** | 1.007 ± 0.1331 | 0.09882 ± 0.08048^****^ | 0.04277 ± 0.02364^****^ | 0.07791 ± 0.06364^****^ | 1.037 ± 0.3038 | 0.2295 ± 0.1442^****^ | 0.1103 ± 0.01965^****^ | 0.1242 ± 0.05791^****^ | 1.023 ± 0.2776 | 0.4284 ± 0.1371^****^ | 0.09604 ± 0.08327^****^ | 0.03076 ± 0.01798^****^ |
| **TGF-β1** | 1.031 ± 0.2795 | 0.3202 ± 0.1551^****^ | 0.08492 ± 0.01603^****^ | 0.04754 ± 0.03975^****^ | 1.051 ± 0.3381 | 0.3242 ± 0.03924^****^ | 0.1711 ± 0.09883^****^ | 0.03157 ± 0.03288^****^ | 1.006 ± 0.1426 | 0.1222 ± 0.04363^****^ | 0.007604 ± 0.003791^****^ | 0.02235 ± 0.004960^****^ |
| **α-SMA** | 1.016 ± 0.2208 | 0.05516 ± 0.007604^****^ | 0.05698 ± 0.05622^****^ | 0.07791 ± 0.06364^****^ | 1.030 ± 0.2701 | 0.2612 ± 0.03988^****^ | 0.1164 ± 0.01211^****^ | 0.01742 ± 0.005416^****^ | 1.008 ± 0.1570 | 0.05927 ± 0.01631^****^ | 0.02025 ± 0.01922^****^ | 0.04763 ± 0.02449^****^ |
| **TNF-α** | 1.054 ± 0.3508 | 0.04666 ± 0.01735^****^ | 0.03194 ± 0.02067^****^ | 0.1018 ± 0.05228^****^ | 1.006 ± 0.1366 | 0.1416 ± 0.03136^****^ | 0.09279 ± 0.07787^****^ | 0.2063 ± 0.07825^****^ | 1.027 ± 0.2931 | 0.02566 ± 0.02112^****^ | 0.03945 ± 0.02176^****^ | 0.04943 ± 0.04861^****^ |
| **IL-1β** | 1.011 ± 0.1560 | 0.1189 ± 0.1091^****^ | 0.05199 ± 0.03760^****^ | 0.2670 ± 0.1480^****^ | 1.031 ± 0.2631 | 0.1342 ± 0.01651^****^ | 0.1229 ± 0.03252^****^ | 0.07391 ± 0.03501^****^ | 1.022 ± 0.2545 | 0.1863 ± 0.1268^****^ | 0.01746 ± 0.007691^****^ | 0.1288 ± 0.04237^****^ |
| **IL-6** | 1.047 ± 0.2901 | 0.1297 ± 0.09505^****^ | 0.04295 ± 0.03688^****^ | 0.1045 ± 0.03923^****^ | 1.022 ± 0.2404 | 0.2630 ± 0.06437^****^ | 0.1618 ± 0.1254^****^ | 0.2417 ± 0.1128^****^ | 1.027 ± 0.2785 | 0.3323 ± 0.1923^****^ | 0.04568 ± 0.03641^****^ | 0.1128 ± 0.02922^****^ |
| **IL-13** | 1.001 ± 0.05524 | 1.319 ± 0.4406 | 3.619 ± 0.2142^***^ | 5.846 ± 0.8173^****^ | 1.002 ± 0.06990 | 2.297 ± 1.033 | 1.380 ± 0.5659 | 1.459 ± 0.5507 | 1.038 ± 0.2879 | 0.7808 ± 0.1495 | 0.3170 ± 0.1801^***^ | 1.436 ± 0.4008 |
| **CCL2** | 1.025 ± 0.2850 | 0.7894 ± 0.4679 | 5.934 ± 1.065^****^ | 2.215 ± 0.6980 | 1.002 ± 0.07248 | 0.5649 ± 0.2959 | 2.072 ± 0.6793^*^ | 1.393 ± 0.3246 | 1.014 ± 0.2064 | 0.6052 ± 0.1611^**^ | 0.6424 ± 0.2783^**^ | 0.05709 ± 0.04742^****^ |
| **p* < 0.05, ***p* < 0.01, ****p* < 0.001, and *****p* < 0.0001 (one-way ANOVA) | | | | | | | | | | | | |

**Table S2.** Rat primer sequences for gene expression analysis

| **Gene** | **Direction** | **Primer sequence** | **Reference** |
| --- | --- | --- | --- |
| **GAPDH** | Forward | 5′-GGC ACA GTC AAG GCT GAG AAT G-3′ | NM_017008.3* |
|  | Reverse | 5′-ATG GTG GTG AAG ACG CCA GTA-3′ |  |
| **α-SMA** | Forward | 5′-ATC CTG ACC CTG AAG TAT CCG ATA-3′ | NM_031004* |
|  | Reverse | 5′-CCA CGC GAA GCT CGT TAT AGA-3′ |  |
| **COL1A1** | Forward | 5′-GAC ATG TTC AGC TTT GTG GAC CC-3′ | NM_053304* |
|  | Reverse | 5′-AGG GAC CCT TAG GCC ATT GTG TA-3′ |  |
| **COL1A3** | Forward | 5′-TTT GGC ACA GCA GTC CAA TGT A-3′ | NM_032085* |
|  | Reverse | 5′-GAC AGA TCC CGA GTC GCA GA-3′ |  |
| **TNF-α** | Forward | 5′-AAA TGG GCT CCC TCT CAT CAG TT-3′ | X66539* |
|  | Reverse | 5′-TCT GCT TGG TGG TTT GCT ACG AC-3′ |  |
| **IL-1β** | Forward | 5′-CAC CTC TCA AGC AGA GCA CAG-3′ | M98820* |
|  | Reverse | 5′-GGG TTC CAT GGT GAA GTC AAC-3′ |  |
| **IL-6** | Forward | 5′-TCC TAC CCC AAC TTC CAA TGC TC-3′ | E02522* |
|  | Reverse | 5′-TTG GAT GGT CTT GGT CCT TAG CC-3′ |  |
| **TGF-β1** | Forward | 5′-CAC CGG AGA GCC CTG GAT A-3′ | NM_021578* |
|  | Reverse | 5′-TCC AAC CCA GGT CCT TCC TA-3′ |  |
| **SMAD3** | Forward | 5′-CGC ATG AGC TTC GTC AAA GG-3′ | NM_013095.3* |
|  | Reverse | 5′-CCG ATC CCT TTA CTC CCA GTG-3′ |  |
| **IL-13** | Forward | 5′-GTG GTC TTG CCA CCC CAG GG-3′ | 4 |
|  | Reverse | 5′-CGC CAG CTG TCA GGT CCA CG-3′ |  |
| **CCL2** | Forward | 5′-AAG AAG CTG TAG TAT TTG TCA CCA AGC TCA-3′ | 5 |
|  | Reverse | 5′-CAT CAG GTA CGA TCC AGG CT-3′ |  |
| *GenBank accession number. | | | |

**SUPPLEMENTAL REFERENCES**

1. Birajdar MS, Kim BH, Sutthiwanjampa C, Kang SH, Heo CY, Park H. Inhibition of Capsular Contracture of Poly (Dimethyl Siloxane) Medical Implants by Surface Modification with Itaconic Acid Conjugated Gelatin. *Journal of Industrial and Engineering Chemistry*. 2020/09/25/ 2020;89:128-138. doi:https://doi.org/10.1016/j.jiec.2020.03.036
2. Hu H, Johani K, Almatroudi A, et al. Bacterial biofilm infection detected in breast implant–associated anaplastic large-cell lymphoma. *Plastic and reconstructive surgery*. 2016;137(6):1659-1669.
3. Galdiero M, Larocca F, Iovene MR, et al. Microbial evaluation in capsular contracture of breast implants. *Plastic and reconstructive surgery*. 2018;141(1):23-30.
4. Paulus P, Holfeld J, Urbschat A, et al. Prednisolone as Preservation Additive Prevents from Ischemia Reperfusion Injury in a Rat Model of Orthotopic Lung Transplantation. *PLOS ONE*. 2013;8(8):e73298. doi:10.1371/journal.pone.0073298
5. Feng C, Wang X, Liu T, Zhang M, Xu G, Ni Y. Expression of CCL2 and its receptor in activation and migration of microglia and monocytes induced by photoreceptor apoptosis. *Mol Vis*. 2017;23:765-777.
